# Advancing human health risk assessment: the role of new approach methodologies

**DOI:** 10.3389/ftox.2025.1632941

**Published:** 2025-10-07

**Authors:** Deepika Deepika, Kanchan Bharti, Shubh Sharma, Saurav Kumar, Rajesh Kumar Pathak, Judit Biosca Brull, Oscar Sabuz, Silvia García Vilana, Vikas Kumar

**Affiliations:** ^1^ Department of Chemical Engineering, IISPV, Universitat Rovira I Virgili, Tarragona, Spain; ^2^ German Federal Institute for Risk Assessment (BfR), Berlin, Germany; ^3^ Center of Environmental, Food and Toxicological Technology (TECNATOX), Universitat Rovira i Virgili, Reus, Spain; ^4^ Escola Politècnica d’Enginyeria de Vilanova i la Geltrú (EPSEVG-GiES), Universitat Politècnica de Catalunya, Vilanova i la Geltrú, Spain

**Keywords:** new approach methodologies (NAM), point of departure (POD), *in-vitro*, omics, *in-silico*, machine learning, data harmonization

## Abstract

New Approach Methodologies (NAMs) hold great potential to fill data gaps for chemicals and modernisation of chemical risk assessment practices. Current toxicity testing is based on conventional approaches with high reliability on *in-vivo* studies, but with time, regulators are trying to move towards *in-vitro* and *in silico* tools enabling efficient risk assessment strategies. Herein, we discuss about different emerging techniques which are or can become a NAM including both *in-vitro* and *in silico* models with particular focus on reducing animal studies and improving decision-making for hazard and exposure assessment. We also discussed about the way to strengthen the regulatory and public confidence in different NAMs and automation of these approaches. Some of these NAMs can help in identifying biochemical mechanisms for toxicity, calculate the point of departure (PoD), develop adverse outcome pathways (AOP), translate risk to multiple species and quantify uncertainty from predictions for multiple chemicals. Scientists and regulators can work together to frame robust guidelines for the practical application of these tools and ensure reproducible results.

## Graphical Abstract

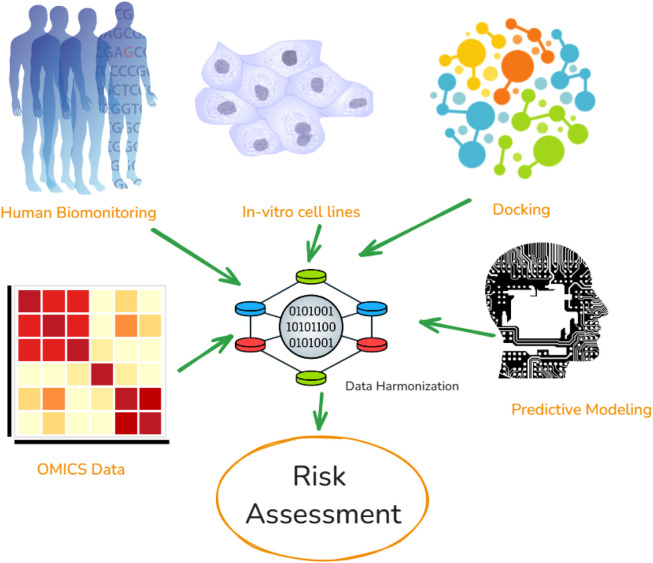

## Highlights

• Contribution of *in-vitro* and *in silico* approaches towards building NAM.
• Automation of different approaches for risk assessment.
• Current limitations in data reporting and steps to improve data fairification.
• Integrating high throughput approaches for risk assessment.

## 1 Introduction

Development of alternative approaches based on the 3Rs principle; reduce, replace, and refine which provides the framework for minimizing animal use in scientific testing has led to different visions for improving toxicological risk assessment, like “toxicology for 21st century”, and “new approach methodologies (NAMs)” for chemical safety risk assessment (Bearth et al., 2025; Sewell et al., 2024; van der Zalm et al., 2022). Currently, there are more than ten thousand chemicals present in the market with thousands of them lacking relevant data for meaningful risk assessment. Testing that vast number of chemicals will take decades to generate enough data, and additionally conducting animal tests are too expensive and ethically difficult, posing challenges to solve the issue of assessing risk from these chemicals to humans and the environment (Sewell et al., 2024). Hence, there is a clear need for NAMs, which can help in filling the existing gaps and accelerate the process of risk assessment.

NAMs in simple words can be defined as emerging technology, methodology, approach, or combination thereof, having the potential to improve risk assessment for fulfilling critical information gaps and avoid or reduce the reliance on animal studies (Kavlock et al., 2018). Regulators like USEPA (United States Environmental Protection Agency), EFSA (European Food Safety Authority), ECHA (European Chemical Agency), and other related agencies have been developing frameworks to implement and use NAMs for regulatory applications (Di Nicola et al., 2023). Integrated approaches for testing and assessment (IATA) combine different data sources, to conclude on the toxicity of chemicals and often include data from NAMs. According to the OECD, an IATA combines multiple sources of information for hazard identification, hazard characterization, and chemical safety assessment. IATA frameworks integrate and weigh all relevant existing evidence, while also guiding the generation of targeted new data when needed, to support regulatory decision-making on potential hazards and risks (OECD, 2016). For simple endpoints or local effects, animal studies can be replaced with *in-vitro*, in-chemico, or *in silico* approaches, however, for complex endpoints, in the current scenario the need for animal studies can be reduced, not eliminated. These methods as NAMs provide data to be used within IATA frameworks. However, by using a comprehensive “weight of evidence” approach and leveraging emerging NAMs there is fair possibility that by weighing and integrating different types of available information decision making on complex endpoints can also be taken without conducting additional animal studies. Further, NAMs can be used to inform population variability, by identifying susceptible populations especially pregnant females, infants, and occupational exposure workers by considering individual health risk and developing regulatory limits for narrow toxicological index chemicals (Di Nicola et al., 2023; Kavlock et al., 2018).

Apart from providing data for risk assessment, NAMs can also help in prioritization, especially supporting grouping approach or read-across. For instance, Point of Departure (PoD) refers to the dose at which a biological response is first observed and serves as the basis for extrapolations in risk assessment (Sturla, 2018). A PoD can be derived from different metrics, including the no-observed-adverse-effect level (NOAEL), lowest-observed-adverse-effect level (LOAEL), benchmark dose (BMD) (Izadi et al., 2012). PoDs can also be estimated using NAMs, which can help in setting safety values when data are limited. This approach can reduce the need for high-tier testing that is currently required to reach conclusions in risk assessment (Levorato et al., 2019). Another approach can be the utilization of publicly available data from ToxCast and toxicogenomic to prioritize chemicals based on bioactivity calculated through *in silico* models (Farmahin et al., 2017). These data can also be used to enrich Adverse Outcome Pathways (AOPs), which describe a sequence of causally linked biological events leading to an adverse health or ecotoxicological effect, and serve as a central framework for mechanistic risk assessment (Ankley et al., 2023; Villeneuve et al., 2014). Computational methods are therefore an integral component of NAMs, as they enable the integration and interpretation of large datasets for hazard and risk assessment. Importantly, computationally approaches have been demonstrated to have clear cost-effectiveness by enabling rapid, large-scale screening compared to resource-intensive animal bioassays and have already been adopted in regulatory contexts such as the U.S. EPA’s Endocrine Disruptor Screening Program and Toxic Substance Control Act prioritization efforts. International agencies including ECHA, Health Canada, and the European Food Safety authority (EFSA) have also used these data, reflecting the growing regulatory acceptance of computational toxicology approaches for chemical risk assessment (Thomas et al., 2019).

**FIGURE 1:**
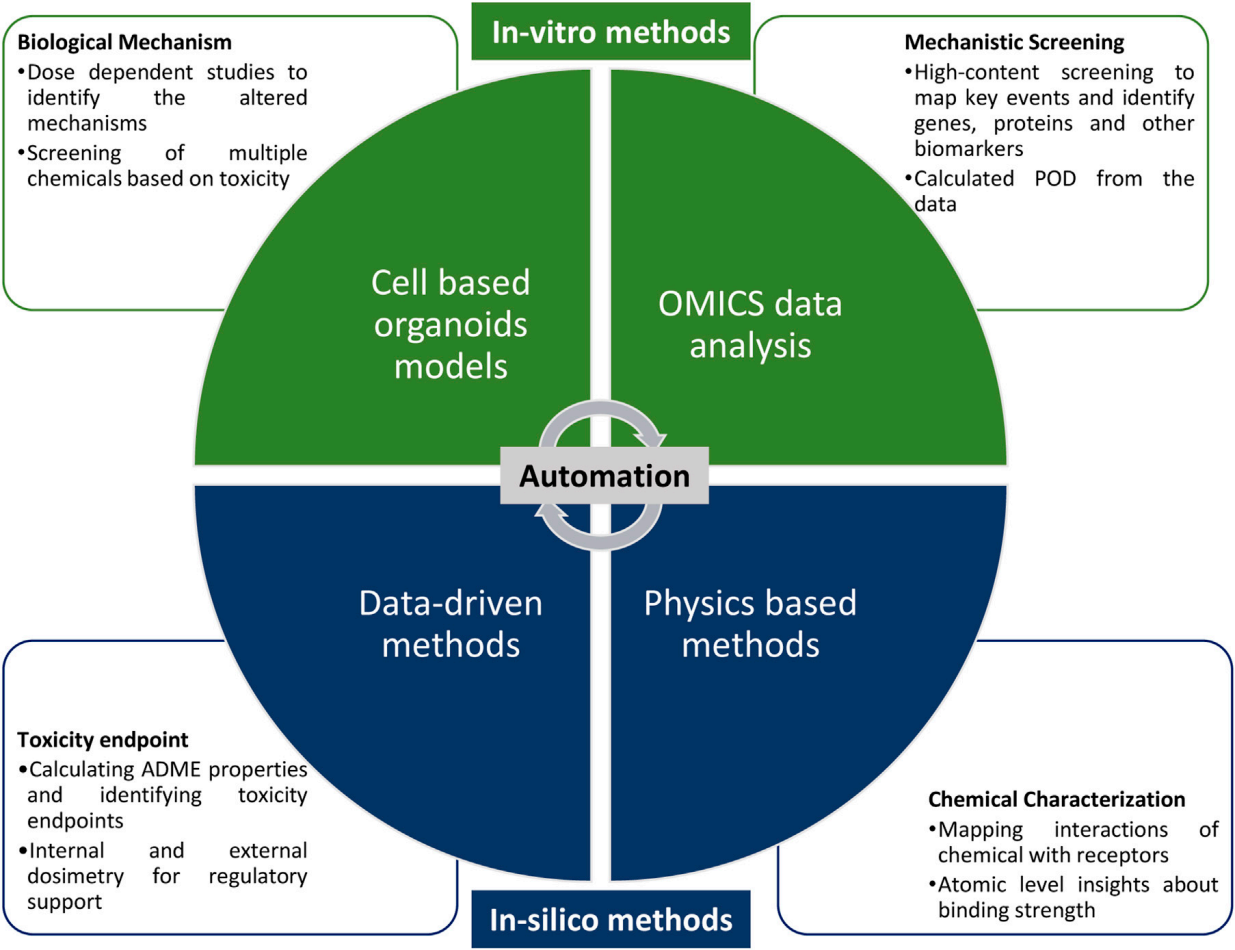
Different approaches which can be utilized for developing New Approach Methodologies (NAMs), along with automation for supporting chemical risk assessment.

The objective of this review is to provide an overview on NAMs being used by risk assessors all over the world, highlight their limitations and challenges, discuss newly emerging NAMs which can help in environment and human health risk assessment in field like, high-throughput *in-vitro*, OMICS analysis, physics-based (molecular docking, molecular dynamic simulations, and density function theory), and data-driven methods (quantitative structure activity relationship: QSAR, physiologically based pharmacokinetic model: PBPK, systems biology models) (Figure 1). In addition, the review addresses approaches to promote the acceptance of these NAMs, including the role of AI/ML in their automation, and discusses the harmonization and FAIRification of data generated by NAMs, with emphasis on integrating evidence from *in vitro* and *in silico* studies to support AOPs.

## 2 NAMs in chemical risk assessment

Integration of *in-vitro* and *in silico* approaches can be used to assess risk and identify the endpoints for regulatory purposes. *In vitro* approaches like 3D cell lines, organoids, spheroids, and microphysiological (MPS) systems are increasingly becoming famous and gaining acceptance by the researchers and toxicologists worldwide. But the OECD framework for the generation of data using *in-vitro* pipeline is still quite blurry, often limiting its usage for regulatory submissions. However, with increased focus on non-animal-based approaches by regulators, the translation of risk through *in-vitro* and *in silico* will become the standard framework in near future (Madden et al., 2020). *In-silico* based approaches can be divided into two categories, one being physics-based cheminformatic with the potential to use at early stages for extracting valuable insight about the chemical and the other being data driven models which can be used at later stages to translate the risk for humans. Techniques like molecular docking can be used to predict binding affinities and can help in prioritizing compounds with toxicity properties. Combining the initial data from cheminformatics to *in-vitro* or integrating multiple *in silico* models can help in building weight-of-evidence for newer and existing molecules.

Novel approaches like the OMICS pipeline, along with benchmark dose modelling (BMD) have the potential to reveal common biochemical response pathways and calculate different limit values like IC50 (Beale et al., 2022). However, due to different types of data being generated, standardisation, data quality, and interpretation are still the bottleneck for acceptance by regulators. The OECD OMICS reporting framework (OORF) has been developed to ensure the reproducibility and quality of data. It can be further used for calculating the PoD and as well combined with other *in silico* tools like PBK model for translation of risk (Farmahin et al., 2017).

Currently, PBK models are being used for risk translation, exposure reconstruction, chemical-chemical interaction, and much more to predict the fate of chemicals in living organisms (8,9). Toxicokinetic tools like httk, TK-plate, and others are gaining popularity and are continuously being improved to increase the incorporation into risk assessment. EFSA used a PBK model to calculate the tolerable weekly intake (TWI) for 4 PFAS considering immunotoxicity as the endpoint (Schrenk et al., 2020). Similarly, other *in silico* tools like QSAR for identifying chemical structural features with relevance for human and environmental hazard, systems biology models for evaluating toxicodynamics can support risk assessment and NAMs approaches. OECD toolbox currently uses a combination of multiple NAMs approaches like *in-vitro*, OMICS, PBK, QSAR, etc. to build weight of evidence for different chemicals and endpoints (Figure 1). Guidance for read-across and structurally similar compounds has also been published to support genotoxicity hazard by EFSA and ECHA (Henriquez et al., 2024). Read-across is an approach in which data from well-studied chemicals are used to predict the properties or toxicity of structurally or mechanistically similar chemicals, thereby filling data gaps while reducing reliance on animal testing. Recently, ECHA has recommended various *in silico* methods like nanoQSAR, grouping and read-across, PBK modelling, molecular dynamics simulations, and adverse outcome pathways for the prediction of risk posed by nanomaterials to humans and the environment (ECHA, 2022). Additionally, techniques like text mining and natural language processing support the curation of information from knowledge sources and facilitate hypothesis development for risk assessment. Implementations and advancement in QSAR, read-across, adverse outcome pathway (AOP), and integrated approach to testing and assessment (IATA) approaches, are always recommended by regulators for toxicological risk assessment (Table 1). Many of these approaches are already being accepted and used by pharmaceutical industries, but the regulators need to develop proper guidelines about using these tools considering their strengths and limitations for improving risk assessment (Table 1).

**TABLE 1:** Different NAM approaches with strengths and limitations for acceptance in risk assessment.

Models	Methods	Strength	Limitation for regulatory acceptance	Strategies for mitigation
*In vitro*	2D and 3D cells	Study of specific molecular mechanisms Mimics tissue architecture and cell-cell interaction Mimics the microarchitecture and physiological responses of human organs	Under-representation of the target organ Lesser cell density than the actual tissue Monoclonal origin of cells does, impairs intracellular signaling	Production of reproducible and high-quality scientific data by following the six principles of Good Cell and Tissue Culture Practice (GCCP)
	OMICS	Identifying significant genes, proteins and metabolites involved in interfering with processes Significant pathways being altered at molecular level	Lack of standardization Lack of transparency in data processing from raw data to an interpretable result Broad in scope and generate data that may be applicable to a wide range of toxicological endpoints	Use of OORF for reporting to ensure critical details on study design, data quality, and regulatory relevance to enhance reproducibility
In silico	Molecular Docking	Calculating binding energy of chemicals with receptors Stability and reactivity of toxic compounds Determination of LD50 by integrating it with deep learning	Inadequacy in scoring function and algorithms can compromise the results	Post-processing of docking results can provide more accuracy in the docking results
	MDS	Can simulate the behaviour of macromolecules	Every change in system due to quantum mechanics cannot be simulated due to approximations Understanding of the complete interaction is not possible because of the very short scale of time step	Longer simulation time can provide better outcome Use of different optimized/designed force fields on the basis of specific contaminants A OECD reporting guideline can be helpful during regulatory filling where MDS has been used
	DFT	Predict the reactivity and stability of compound	Empirical force fields cannot capture bond breaking and are limited by parametrization, advances such as reactive force fields	QM/MM hybrid methods, and polarizable force fields are making MD both more accurate and more broadly applicable for toxicological studies
	QSAR PBK	Enables prediction of chemical properties or bioactivities by linking molecular structure to experimental outcomes, allowing efficient design of new compounds without extensive testing Estimating human exposure using IVIVE-based mechanistic PBK models, with the potential to integrate toxicokinetic with AOPs by utilizing *in vitro* assay results to quantify AOPs	Lack of acceptance threshold in the assessment element such as applicability domain fit, reliability scoring, or goodness-of-fit criteria Limited validation datasets due to unavailability of PK data of many chemicals	Ensuring predictive models are built on high-quality, well-characterized experimental data, including details on test variability, potential confounding factors, and chemical purity to enhance model reliability and structure-effect correlation Following OECD PBK and other reporting template while building models

### 2.1 *In vitro* methodologies

The field of *in-vitro* models is evolving rapidly and has become crucial for toxicology and chemical assessment (Wang et al., 2024). These models have gained popularity, especially after the implementation of the 3Rs (replacement, reduction and refinement) principle, as they offer a robust alternative to animal testing. In the section below, we will be discussing about advanced biological system which carries the potential to become NAM.

#### 2.1.1 2D to 3D cell culture and organoids

Traditional *in-vitro* models mostly rely on 2D configurations utilizing animal and human cell lines. These models have been widely used because of their simplicity and cost-effectiveness, but they fail to capture the complexity of human physiology, including tissue architecture, cell-cell interactions and metabolic capacity (Wang et al., 2024). This limits their predictive value for human health outcomes. To address these limitations, a wide range of more advanced models has emerged.

The 3D cultures, organoids derived from stem cells, and microphysiological systems (MPS) such as organ-on-a-chip platforms better mimics tissue architecture (Cacciamali et al., 2022). These systems, more accurately, stimulate the complexity of human tissue and its interactions, allowing for investigations of organ-specific toxicity, barrier integrity, and multicellular responses to chemical exposures (Duval et al., 2017; Ravi et al., 2015). Since responses to substances can differ significantly across species, these models also aid in identifying diseases that might not be evident in animal models (Loewa et al., 2023). To provide a clearer understanding of *in-vitro* models applicability in chemical risk assessment, examples of these emerging models are presented, highlighting both their potential and limitations.

3D culture particularly organoids, have been used to study the toxic effect of different substances, such as pesticides, microplastics, or particulate matter, on different organs. Although being simpler than the whole organ, they exhibit fundamental features such as cellular organization, differentiation and interaction (Wan et al., 2024). For example, liver organoids have been applied to study metabolism-mediated hepatotoxicity, while brain organoids have been used to investigate neurodevelopmental toxicity. A review conducted by Cong et al. summarized the recent advances in relation to chemical risk assessment, concretely they recap the toxicity of microplastics and other environmental compounds in a wide variety of organoids (Cong et al., 2024).

In accordance with the examples mentioned above, Cheng et al. (2022), Cheng et al. (2023) demonstrated that liver organoids recapitulate hepatotoxic and lipotoxic effects induced by microplastics, alone or in combination with BPA. Through evaluations of cellular toxicity, lipid metabolism alterations, oxidative stress or inflammatory pathways, they predicted the potential risk of microplastics in liver fibrosis and cancer, highlighting the utility of organoids to assess chemical interactions. Similarly, brain organoids treated with organophosphate pesticides or particulate matter have been observed to produce autism-related disorders by reducing the CDH8 protein and affecting neuronal growth. In addition, exposure to diesel particulate matter was associated with altered mitochondrial function, while exposure to a common additive in plastic products reduced neuronal proliferation and migration, and induced apoptosis in brain organoids, further demonstrating the value of organoids in revealing specific mechanism of chemical toxicity (Albanese et al., 2020). Although organoids show great potential in toxicity assessment, their ability to stimulate complex pharmacokinetic processes is still limited. For example, they cannot fully replicate the blood-brain-barrier, which is crucial for evaluating drugs intended for central nervous system (CNS) disease (Wan et al., 2024).

In the same line, to better capture organ complexity MPS, such as organ-on-a-chip systems, have been developed. These microfluidic devices mimics the mechanical and biochemical environment of human organs, allowing multi-organ systems with diverse cell co-cultures and 3D tissue structures connected by microfluidic channels that replicate blood vessels (Bhatia and Ingber, 2014). Among the different application of these models, they have revolutionized respiratory disease research and have opened new possibilities for studying kidney function (Ashammakhi et al., 2018; Shrestha et al., 2020). A recent study, which evaluate nanoparticles toxicity using a lung-on-a-chip model demonstrated that organ-on-a-chip systems have a greater accuracy in assessing adverse effects compared to animal models (Shrestha et al., 2020). Specifically, Shrestha et al., found that nanoparticles exposure increased the expression of the intercellular adhesion molecule 1 and the production of reactive oxygen species. In addition, lung-on-a-chip models allowed the study of nanoparticles transport, demonstrating an acceleration of the toxic effect on lung, underscoring the importance of mechanical movements on toxicity assessment (Shrestha et al., 2020). Similarly, kidney-on-a-chip models are potentially relevant for chemical risk assessment, due to this organ is the main way of excretion of environmental compounds (Lepist and Ray, 2016). Li et al. found that cadmium exposure in a kidney-on-a-chip device with glomerular endothelial cells declined cell-viability and increased endothelial injury by increasing LDH in a dose-dependent manner. Consequently, a reduction in the tight junction protein was also observed, indicating that cadmium disrupt barrier integrity and increase permeability depending on the dose administered (Li et al., 2017). These studies help to understand the alterations produced by environmental chemicals that modify their absorption or excretion, making them more aggressive.

Other new advanced *in-vitro* approaches, particularly tissue engineering, have shown promising applications for chemical risk assessment (Cui et al., 2018). This strategy enables the creation of functional tissue models such as skin, bone, skeletal muscle and cardiovascular systems, which can be used for drug screening, toxicity testing and disease modeling (Mirshafiei et al., 2024). For instance, mesenchymal stem cells (MSCs) have been applied to regenerate bone tissue, differentiating it into osteoblasts with specific growth factors such as TGF-β, TGF-α or HGF, providing platform to assess compound-induced effect on bond formation and scaffold integration (Stamnitz and Klimczak, 2021). Similarly, human PSCs-derived cardiomyocytes, when combined with extracellular matrix scaffolds, can mimic myocardium contraction and electrical function, offering a model to evaluate cardiotoxicity of chemicals (Jackman et al., 2015). Despite these advances and their high potential for risk assessment, tissue-engineered systems remain in development, and challenges such as vascularization, scalability, and standardization of bio-ink still need to be addressed to facilitate its broader applications in chemical risk assessment (Cui et al., 2018).

Despite these advances, regulatory uptake of these methods has been limited due to challenges in validation, reproducibility and harmonization. Several OECD guidelines already incorporate *in-vitro* approaches which have been approved such as OECD TG 439, 487 and 496 used for the hazard identification of irritant chemicals or those with the potential to induce serious eye damage by the reconstruction of human epidermis or cornea, as well as genotoxicity test (OECD, 2021b; OECD, 2023; OECD, 2024). However, addressing complex endpoints such as repeated-dose toxicity, carcinogenicity or reproductive toxicity remains a significant challenge in the validation and implementation of *in-vitro* models (Miccoli et al., 2022; Schmeisser et al., 2023). Further progress will depend on standardized protocols, international validation efforts, and the integration of different methodological domains (ECHA, 2022; Schmeisser et al., 2023; Perkins et al., 2022).

#### 2.1.2 *In vitro* data for hazard assessment

Many laboratories around the world are working to develop and optimize alternative *in-vitro* test methods for hazard assessment of compounds for either pre-screening of compounds or to identify toxicity and mechanism of action. Many developed *in-vitro* models as well as high throughput techniques like imaging, high content screening etc. have the potential to reduce cost and animal usage but the validation of such models and techniques is complex and challenging and it takes years for such approaches to get official regulatory acceptance. Nonetheless, there have been a lot of examples in recent years with pre-validated and validated methods related to genotoxicity, developmental neurotoxicity, safety testing in cosmetic products and much more to partially or fully replace animal testing for hazard assessment.

One good example is OECD guideline 493 which is well characterized, validated and accepted providing *in-vitro* procedure for identification of skin irritants. Reconstructed human epidermis composed of epithelial cell layers is recommended along with validated test method for cell viability. As per the regulation 1907/2006 for REACH, this alternative testing can be used for skin irritation and corrosion for substances allowing complete replacement of animal experimentation (Bas et al., 2021). Data shows that non-animal test methods have increased three times for skin corrosion/irritation, four times for eye irritation/damage and twenty times for skin sensitization for period 2017–2019 under REACH (Burden et al., 2021). There is also a lot of effort ongoing for developmental neurotoxicity NAM incorporating high content imaging (HCI) for apoptosis, proliferation, synaptogenesis and neurite outgrowth. Carsten et al. in his work evaluated a dataset of 92 chemicals for 57 assays including both microelectrode array, network formation assay (NFA) and HCI assay relevant to DNT. They found that NFA along with HCI can help in assessing key functional processes in neuronal development which is a good starting point for classifying DNT positive and negative chemicals. To improve classification of DNT NAM evaluation chemicals, addition of DNT NAM assay to integrated screening paradigm should include both neuronal and non-neuronal cell types which should represent important neurodevelopmental processes like neuronal crest cell migration and myelination that are not included currently (Carstens et al., 2022). Genotoxicity is another area where *in-vitro* test methods are advanced and accepted by regulators (OECD TG 471, 473, 476, 479, 481, 482, and 487). But, still many of alternative *in-vitro* test methods are not fully accepted by regulators due to lack of validation. Test method validation refers to a process based on scientifically sound principle by which relevance and reliability of a particular approach, method, process or test are established for a specific purpose (Kandárová and Letašiová, 2011). In brief, any *in-vitro* test method, whether new or updated, needs to be relevant and reliable, i.e., validated to be accepted by regulators. Establishing this aspect is a critical step in validation for NAM. Often, comparison of data generated using *in-vitro* test methods between laboratories and within labs is challenging as most researchers do not work with standardized and detailed protocols. Implementing standard operating procedures (SOP) in the laboratory for tests can help to achieve harmonization and also help with validation and regulatory acceptance of the test methods. Also, the concept of IATA in which different test methods are combined to predict one end point is challenging as there are multiple test guidelines. A successful example of such approach is endpoint for human skin sensitization where IATA is based on AOP describing the linkage between chemical interaction with a biological system at molecular, subcellular, cellular, tissue and organ level (Bas et al., 2021).

#### 2.1.3 OMICS analysis

It is important to note that, in addition to demonstrating toxic effects, *in-vitro* models allow the acquisition of mechanistic information like the interaction between molecules, alterations in cell metabolism or the specific functions of each organ, understanding how chemical perturb biological pathways. This is essential for establishing safety thresholds and relevant human PoD (Zhang et al., 2018). Therefore, by integrating these advanced *in-vitro* systems with other approaches such as OMICS, it becomes possible to link cellular responses to AOPs and derive quantitative safety information, enhancing their relevance for chemical risk assessment (Perkins et al., 2022). In addition, integrating these innovative technologies with *in silico* approaches like IVIVE based PBK modelling can help in improving risk assessment (Kreutz et al., 2024; Zhang et al., 2018). This strengthens their regulatory relevance and highlights how *in-vitro* models, when combined with complementary techniques enhances our understanding of the biological processes underlying human exposure to chemicals.

The use of NAMs in the context of 3D cell cultures, MPS, and organoids presents a promising future for chemical risk assessment, providing more accurate, ethical, and human-relevant alternatives to traditional animal models. Although these *in-vitro* models have potential, they remain limited in their ability to reproduce human physiology and predict systemic toxicity (Schmeisser et al., 2023). To maximize their usefulness, NAMs must be standardized and validated for their regulatory acceptance and complemented with analytical approaches that provide specific information at the molecular level. To this end, OMICS is a potential new tool that allow us to capture cellular and molecular responses in our models. Combining *in-vitro* models with these techniques improves the interpretability, reproducibility and regulatory relevance of *in-vitro* models.

OMICS technologies provide the essential molecular and cellular readings needed to turn *in-vitro* systems into robust tools for chemical risk assessment. Toxicogenomics approaches, in particular, can help in characterizing human risk and have the potential to become NAMs by analyzing the toxicity of the chemicals at the molecular and cellular level. In the broader aspect, OMICS encompasses genomics, transcriptomics, metabolomics and proteomics study which can identify changes in the genotype and phenotype of the organism and relate them to the AOPs (Addicks et al., 2023).

Transcriptomics data obtained from cell lines can be used to identify activation of specific targets and also to calculate PoD based on changes in gene expression as an approach to screen large number of chemicals. This can identify molecular initiating events (MIE) and key events (KE) within the AOP to understand the chemical’s mode of action based on targeted differential gene expression identification (Harrill et al., 2024). Multiple studies used transcriptomics data for risk assessment. For example, Matteo et al. analyzed BPA and 15 data poor alternatives and their effects on human breast cancer cell lines to analyze the toxicity using the BMD and PoD analysis (Matteo et al., 2023). Similarly, Addicks et al., evaluated the effects of Per- and polyfluoroalkyl substances (PFAS) on the human liver spheroids using the transcriptomic PoD (t-PoD) (Addicks et al., 2023). This, high-throughput transcriptomics data allows us to look for responses across multiple molecular pathways compared to traditional *in-vitro* data. Although, multiple approaches have been applied to calculate molecular level PoD, there is no consensus on t-POD, primarily due to its variability across cell types and experimental conditions.

Metabolomics includes identifying and quantifying the complete set of metabolites whereas proteomics identifies and characterizes the entire set of proteins providing a holistic understanding of cellular response. Malinowska et al., illustrated integration of *in-vitro* metabolomics with high-throughput screening to support decision-making in risk assessment of chemicals using HepaRG cells and CdCl_2_ as a case study (Malinowska et al., 2022). Viant et al., demonstrated the usability and reliability of *in-vivo* metabolomics study of 8 chemicals in rats to group them based on the activity of the metabolites ensuring to make the analysis FAIR and more reproducible, following guidelines from OECD and ECHA, and reflect the need to establish standard quality control steps for processing the data and improving risk assessment (Viant et al., 2024).

To improve accuracy and overcome the limitation of averaging signals across heterogeneous cell population leading to false discoveries, single-cell omics techniques are gaining attention to provide better insights into the complexity of the tissue of interest. This can provide information about how chemicals affect specific cell types or pathways, identify rare toxicologically relevant cell populations, and enable molecular phenotyping. However, one of the challenges in this study is isolation of cells from whole tissue before analysis, which employs more complex and costly techniques like Magnetic-activated cell sorting (MACS), Fluorescence-activated cell sorting (FACS), cell barcoding and various microfluidic techniques. To address this limitation, several platforms have been developed (Gierahn et al., 2017). Single-cell omics have a wide range of applications in cancer-research, immunology, and developmental biology. Wu et al., used single-cell RNA sequencing and multi-omics analyses to characterize a novel signature comprising 33 genes related to tobacco carcinogens to elucidate the progression of bladder cancer through fibroblasts-induced immune invasion and epithelial mesenchymal transition (Wu et al., 2024).

In spite of obtaining accurate information with the single-cell omics technique, in response to toxicants, multimodal single-cell omics should be used effectively to understand cellular behavior and regulation, as well as to correlate findings with toxicity pathways and AOPs. An advanced approach like spatial transcriptomics provides insights into the spatial distribution of changes in gene expression in the tissue and captures the specific position of changes in the transcriptome of the tissue sample. The combination of single-cell omics strategies and spatial transcriptomics can be very useful in identifying the organ-specific toxicity, which can help in AOP identification and related biomarkers discovery with more precision. Chen et al., analyzed the progression of cerebral malaria in the murine brain and its treatment with artemisinin using spatiotemporal profiling, which demonstrates the potential of the technique to be used in risk assessment of the chemical (Chen et al., 2025).

Currently, the major challenge in omics analysis is data analysis and integration of data generated from multi-omics techniques, Since the dimension of data generated is different, Multi Omics Factor Analysis (MOFA) (Argelaguet et al., 2018) and Regularized Generalized Canonical Correlation Analysis (RGCCA) (Tenenhaus and Tenenhaus, 2011) are two of the promising algorithms that can be used for the integration of multi-omics datasets. A combination of multimodal single-cell omics analysis and one of these algorithms may help toxicologists with better risk assessment of the chemicals. For the data analysis of omics data, preprocessing is a crucial step for the quality of the analysis, which should be uniform and robust for better reliability of the results for the regulatory bodies. OECD provides a framework called OECD Omics Reporting Framework (OORF) for reporting elements for the regulatory use of omics data from laboratory-based toxicology studies. The main aim for developing this framework is to facilitate data sharing and promote reproducibility in omics toxicology to make it more harmonized and standardized. It provides guidelines for reporting different microarray data: RNA-seq, qRT-PCR, NMR metabolomics, and proteomics along with data analysis and experimental designs. It also provides standard preprocessing steps for the analysts to be followed for improved analysis and minimum false discovery rate (OECD Omics Reporting Framework, 2023).

### 2.2 In-silico methodologies

#### 2.2.1 Physics based methods

These methods are frequently used in drug discovery but is less utilized in NAMs for human health risk assessment. In recent years, computational chemistry approaches such as molecular docking, density functional theory (DFT) calculations, molecular dynamics simulations, and binding energy calculations using Molecular Mechanics Poisson–Boltzmann Surface Area (MM/PBSA) and Molecular Mechanics Generalized Born Surface Area (MM/GBSA) have gained significant attention as several studies have been published in environmental science journals (Trisciuzzi et al., 2018; Zheng et al., 2025). These approaches help predict the interactions between hazardous chemicals and human receptors, providing insights into their binding mechanisms.

Molecular docking is a technique in computational chemistry which elucidate the interaction between a ligand and a receptor complex by simulating the spatial and energy matching between ligands and receptors. Through this the mechanistic of the toxicological effect due to any chemical can be understood. Through this technique large scale screening of chemicals based on their affinity to a specific target can be done. By providing information on binding energies, interaction residues and interaction patterns, Ortega-Vallbanaet et al. demonstrated that tools like DockTox can facilitate the virtual screening of small compounds that target MIE-associated proteins (Ortega-Vallbona et al., 2025). This approach hence has potential to inform AOPs and mechanistic understanding in risk assessment. Advances such as semi-empirical quantum charge calculations like PM6 have improved accuracy in pose prediction, particularly for charged systems and metalloproteins (Bikadi and Hazai, 2009). Importantly, docking can generate hypotheses about potential toxicological targets when experimental data are lacking, supporting the reduction of animal testing and aligning with the 3Rs principle. The potential of the method can be validated in the fact that EFSA in its scientific opinion document also suggest the use of docking for grouping of multiple chemicals based on structural similarity considering multiple features like class of chemicals, presence of functional groups, breakdown products or similar precursor to increase the confidence in the assessment (More et al., 2021). However, the accuracy of results depends heavily on the quality of input structures, and docking programs/algorithms used for the purpose. They generate binding poses that resemble experimentally determined structures; however, challenges remain in accurately reproducing ligand conformations and in accounting for protein flexibility, since most docking approaches still treat the receptor as rigid proteins, which reduces reliability when conformational flexibility is essential. Apart from this no single program deems fit for all the targets (Warren et al., 2006). False positives are common and docking predictions are rarely sufficient as standalone evidence. A critical limitation arises from the reliance on scoring functions as they rely on large approximations to simplify complex molecular recognition processes, which introduces inaccuracies and reduces predictive performance (Cross et al., 2009).

On the other hand, molecular dynamic simulations (MDS) apply Newtonian mechanics to model the movements and interactions of atoms over time. By solving the equations of motion, MDS enables investigation of protein flexibility, conformational changes, and stability during ligand binding. These simulations provide valuable insights into the dynamic behaviour of proteins and their complexes, offering a mechanistic understanding that is critical for both elucidating biological function and supporting chemical risk assessment. Beyond structural dynamics, MDS can quantify conformational stability, residual mobility, compactness, solvent-accessible surface area (SASA), essential dynamics, and Gibbs free energy (FEL) analysis. FEL analysis helps identify high- and low-energy transition states and visualizes the dynamic behaviour of the system. Furthermore, MM/PBSA and MM/GBSA calculations use molecular dynamics simulation data to estimate binding energy components such as van der Waals, electrostatic, polar solvation, SASA, and total binding energy (ΔG) of toxic chemicals with estrogen related receptor gamma (Pathak et al., 2024).

Density Functional Theory (DFT) can be used to calculate the highest occupied molecular orbital (HOMO) and lowest unoccupied molecular orbital (LUMO) energies. The HOMO-LUMO gap is an important parameter for determining the stability and reactivity of toxic chemicals (Pathak and Kim, 2024). Apart from these characteristics DFT can also provide quantum-chemical descriptor such as atomic charge distributions, which when combined with energy gaps and stability profile captured the mechanistic aspects of metabolic activation and have been successfully applied to predict Ames mutagenicity of primary aromatic amines (Kuhnke et al., 2019). Conceptual DFT has also been successfully applied in screening of emerging pollutants where a rule of thumb was provided for differentiation between pollutants. The study gave evidence about the bioaccumulation of pollutant to be “too hard” which binds with protein e.g., PFAS compounds and “too soft” which binds with lipids. This approach overcame the barrier of the non-availability of experimental data where computational method can be applied to decipher the properties of any chemical (Li X. et al., 2024). These approaches can find their value as NAM since they provide atomic-level insights into the binding strength of chemicals with their target receptors in humans. They bridge the gap between hazardous chemicals and their receptors by revealing key interactions, facilitating the identification of hazardous chemicals with varying affinities for specific targets. This information can support risk assessment and aid in developing strategies to mitigate the effects of harmful chemicals by designing competitive inhibitors that disrupt their interaction with human receptors (Jaeger-Honz et al., 2025). Together, they can provide a tiered approach that balances computational cost with predictive power for chemical risk assessment.

#### 2.2.2 Data driven methods

Computational modeling techniques like QSAR, read-across, PBPK, and IVIVE play a pivotal role in toxicology prediction and chemical safety assessment and can be used as an alternative to animal testing for REACH regulation (European Chemicals Agency, 2020).

QSAR/quantitative structure property relationship (QSPR) is one of the methods recommended by REACH for supporting the substance registration process which basically relates the set of descriptors (X) with the response (Y). These models can be used to predict physico-chemical properties of compounds as well as biological activity or toxicity based on molecular structure for newer and existing chemicals. However, to facilitate the consideration of QSAR models for regulatory purposes, it should follow the QSAR modeling reporting framework (QMRF) which can ensure reproducibility and confidence in model predictions. Apart from individual models, platforms like OECD QSAR toolbox is being used by regulators to perform initial screening of chemicals as well as to predict ADMET properties (Yordanova et al., 2019). However, still the data for environmental chemicals is limited compared to the drugs, as a result most QSAR models are trained on drug data, leading to biasness for the prediction of environmental chemicals (Moreau et al., 2022). Additionally, poor performance of QSAR model could be due to multiple factors, including data quality and relevance, choice of descriptors, model complexity, quantification of uncertainties, and ensuring predictions fall within the model’s applicability domain. These aspects should be carefully evaluated by model developers, users, and third-party assessors so that the poor performance of the model can be avoided (Cronin et al., 2025). Importantly, QSAR models not only support hazard prediction but can also generate input parameters that, when integrated with PBK framework, enhance toxicokinetic modelling and translation of *in vitro* data to *in vivo* exposure.

Read-across is an important data-driven approach widely applied in regulatory toxicology, particularly for data-gap filling in chemical risk assessment. It uses information from a source (data-rich) chemical to predict the properties of a target (data-poor) chemical, thereby reducing the need for experimental testing (Read-Across Assessment Framework, 2017). The approach typically relies on showing the similarity of an analogue to a target substance by comparing chemical structure, physicochemical properties, metabolism and toxicokinetics, toxicodynamics, and structural alerts identified through predictive QSAR approaches. Such similarities and differences should be clearly documented, ideally within a data matrix that highlights consistent trends across the category. The robustness of read-across can be strengthened by applying the ECHA assessment framework, which requires a hypothesis-based justification, evaluation of potential contradictions, credible extrapolations, and clear specification of substance identity and composition, including impurities. Collectively, these practices increase transparency and regulatory confidence in read-across predictions (Alexander-White et al., 2022; Read-Across Assessment Framework, 2017).

For toxicokinetics of chemicals, a generic PBK model can be used to predict the concentration-time profile or toxicokinetics of chemicals (Deepika & Kumar, 2023). Currently, QSAR along with PBK model can help in generating parameterization data required for building the model. Open access models like HTTK generate data for parameters like absorption rate constant based on Caco-2 cell lines, metabolic data, protein binding, etc., which helps in reducing dependence on animal studies. OECD PBK model reporting framework includes the guideline for developing confidence in the model with integration of *in-vitro* through IVIVE, QSAR, along with PBK for toxicokinetic prediction, which is a step toward next-generation PBK modeling (NG-PBK) (Paini et al., 2019). Next-generation models can utilize “big data” such as information from high-throughput or high-content *in-vitro* screening assays or omics technologies along with PBK for predictive toxicology and safety assessment (Ram et al., 2022). Quantitative IVIVE (QIVIVE) which combines *in vitro* data with *in silico* methods such as PBK modelling together with information on metabolism, transport, and binding to estimate the likelihood of harmful effects from environmental exposures also plays a key role in NAMs-based risk assessment by translating *in vitro* toxicity data to *in vivo* exposure, aiding chemical prioritization (Moreau et al., 2022). The IVIVE uses *in-vitro* high-throughput biological responses for the prediction of *in-vivo* exposure to estimate the safety threshold for humans. The utilization of PBK enables the consideration of factors like redistribution and metabolism in the results obtained from *in-vitro* test that ultimately translates it into exposure relevant at the organism level (Hines et al., 2022; Najjar et al., 2022). The reliability of QIVIVE-PBK is limited by constraints in clearance predictions, metabolic kinetics, and focus on parent chemical toxicity, necessitating improvements for broader application (Moreau et al., 2022). For instance, in a QIVIVE study predicting nicotine delivery product exposure, the model successfully estimated toxic effects but faced challenges in addressing the potential interaction occurring between the chemicals since modelling and parameterizing could be done only for nicotine (Moreau et al., 2024). In a similar manner another study highlights the challenge of building the QIVIVE model for chlorpyrifos exposure to predict developmental neurotoxicity since the model was limited to multiple oral exposures rather than the real-world exposure scenario, which occurs simultaneously from different routes like dermal and inhalation (Algharably et al., 2023).

Nonetheless, these *in silico* models have potential applications for different regulatory purposes and the potential to become NAMs by facilitating efficient translation of risk. For instance, USFDA supports the use of IVIVE and PBK for filling data gaps to support drug development (Hines et al., 2022). Similarly, these approaches can be used for risk assessment but the clear guidelines about its usage for regulatory purposes are still lacking. HTTK was used to calculate the PoD for 448 chemicals using *in-vitro* bioactivity data (Paul Friedman et al., 2020) and to prioritize chemicals for further screening, supporting the potential of these tools for facilitating interpretation of experimental data.

### 2.3 Approaches for regulatory acceptance of NAMs

NAMs despite having the potential to replace animal experiments usually suffer from the scrutiny of the risk assessors due to either lack of proper validation or huge uncertainty or lack of harmonization. In case of *in-vitro* studies, OECD has published a guidance document (OECD guidance document 211) with the intent to assess the relevance of the large amount of data generated by the alternative *in-vitro* tests which might be of practical value. To check the scientific robustness and context appropriateness, OECD suggest these methods to be reported in a definite framework which should be helpful in evaluating uncertainties and scientific confidence (OECD, 2014). In line to this, to address the problem of overlooking to the test method details, Krebs et al. developed a template called as ToxTemp to follow the requirements of the OECD guidance document 211 which can guide the users to get the information details along with inclusion of acceptance criteria for test elements with sufficient description of cells employed in the study (Krebs et al., 2019). To operationalize this, OECD introduced Harmonised Template 201 (OHT 201) in 2016, a standard format for reporting mechanistic and intermediate effects across NAMs, including *in vitro*, *in silico*, and *ex vivo* methods (Carnesecchi et al., 2023).

According to EFSA, to enhance regulatory acceptance, docking outcomes should be interpreted within a defined applicability domain, integrated with complementary *in silico* approaches (QSAR, ML) under a weight-of-evidence framework, and linked to mechanistic anchors such as MoA or AOPs. Use of standardized open-source tools (e.g., OECD QSAR Toolbox, VEGA) can further increase transparency and reproducibility, thereby strengthening regulatory confidence (More et al., 2021).

In a similar manner OECD guidance document for PBK modeling guides the development of PBK models with their validation to increase their scientific validity. The main objective of this guidance document is to offer a transparent and uniform structure for model assessment in order to facilitate communication between PBK model developers and regulators who evaluate and approve their use (OECD, 2021a). To further streamline the data-driven NAMs OECD has developed its toolbox. The primary advantages of the QSAR Toolbox are its ability to screen for environmental fate endpoints, acute ecotoxicity endpoints, and toxicity endpoints like mutagenicity, skin/eye irritation, and sensitization. Early in the R&D process, the toolbox can also be used to screen possible novel chemicals to find those that are most likely to have a favourable hazard profile. It also has the capacity for clustering chemicals for read-across study as the toolbox incorporates the information and tool from diverse sources into a logical workflow (OECD). A list of discussed NAMs with their strengths, limitations and mitigation strategies has been provided in Table 1.

## 3 Combining *in-vitro* and *in silico* methods with AOP

AOP represents the shift from conventional toxicity testing to more mechanistic framework for establishing disease map or moving to adverse outcome. In general, *in-vitro* test methods by informing the biological pathways, *in silico* models for quantification and prediction of events, and AI methods for data-gap filling along with other experimental data can help in enriching the information for an AOP (Corradi et al., 2022). AOPs have seen a significant growth in last few years with many *in silico* approaches being developed to make them suitable for human risk assessment by considering real-exposure scenarios. In short, an AOP can help in addressing specific endpoints but currently they are more qualitative rather than quantitative which limits their usage for regulatory purpose.

There is already a lot of ongoing work related to quantification of AOPs using *in-vitro* and *in silico* approaches where *in-vitro* assays can provide data on mechanistic end points and *in silico* approaches like Bayesian network, generalized linear or regression model or mechanistic modelling can incorporate the data into quantitative framework to predict adverse outcome. Sewer et al. used data from advanced organotypic airway model exposed to tobacco heating system aerosol or combustible cigarette smoke for AOP 411 which is on decreased lung function due to oxidative stress. They used regression model for key event relationship (KER) using gene expression and other data from *in-vitro* model. Two mathematical modelling-based approach: 1) empirical data based for KER related to oxidative stress, and 2) mechanistic systems biology-based approach for other key events related to AOP 411 was used. Overall, this is a good example of AOP-based approach aligning with IATA principles by integrating *in-vitro* data with computational modelling to predict decreased lung function with aerosol exposure (Sewer et al., 2024).

The huge mechanistic knowledge captured in AOP network (AOPN) can help in the shift towards non-animal and human-based NAM. Vinken et al. showed a framework for NAM to predict hepatotoxicity composed of *in-vitro* systems which can allow tiered testing at transcriptional, translational and functional level for enriching AOP network. *In vitro* systems and a battery of assays mechanistically anchored in an AOP network can be helpful towards NAM development. Some chemical-induced liver toxicity like cholestatic and steatotic drug induced liver injury (DILI) can be predicted by simple *in-vitro* models like monolayer culture of hepatocytes while some liver endpoint needs complex models like MPS (Vinken, 2024).

**FIGURE 2:**
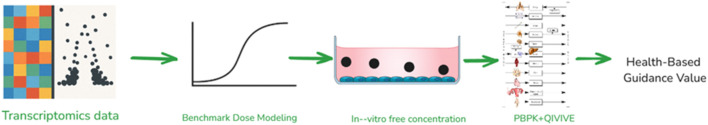
NAM based framework integrating *in-vitro* and *in silico* models for calculating human health-based guidance value.

Another successful example of NAM is where the health-based guidance value was calculated for PFOA using *in-vitro* transcriptomics data (Figure 2). An *in silico* workflow was developed with PFOA as lead molecule comprising of calculating a) PoD for significant genes, b) calculating freely available PFOA *in-vitro*, and c) using QIVIVE for estimating *in-vivo* dose and later tolerable daily intake (Silva et al., 2024). This framework can provide vital information for risk assessment and, by including the uncertainty factor, can further help in reducing the risk and choosing a conservative value for a sensitive population. There are some limitations to this approach. For instance, the relevance of molecular pathway perturbations in predicting apical toxic endpoints, the linkage between molecular and apical changes and guidelines for the uncertainty factor for PoD need to be established (Cattaneo et al., 2023).

NAMs carry the potential to transform risk assessment, especially with the improvement in computational modelling and high-throughput *in-vitro* data. NAM for specific endpoints like skin sensitization, skin and eye irritation, genotoxicity and endocrine activity are sufficient stand-alone with potential to inform about risk assessment. However, there is instance where a battery of NAMs has been used to conclude for the estimation of safety margin for pthalates; di(2-ethylhexyl) phthalate (DEHP) and di-n-butyl phthalate (DnBP) highlighting the value of integrating diverse NAMs within IATA. In this study, the AOP framework was applied to guide the selection of an *in vitro* assay for bioactivity testing, while Aggregate Exposure Pathways (AEPs) were developed to capture critical exposure information. These exposure data were then incorporated into a PBK model to predict internal metabolite concentrations. Finally, the model outputs were combined with in vitro-derived relative potency factors to calculate an internal margin of safety of ∼13,000 (Clewell et al., 2020). In a similar manner, a read-across case study on thirteen branched aliphatic carboxylic acids demonstrated how multiple NAMs can be integrated to strengthen regulatory confidence in data-gap filling. Structural similarity served as the starting point, but biological similarity was established using *omics* approaches—specifically, transcriptomic analysis of differentially expressed genes in human HepG2 liver cells. An AOP network for hepatic steatosis guided targeted *in vitro* testing of MIEs and KEs across multiple human liver models (HepG2, HepaRG spheroids, primary human hepatocyte), complemented by functional assays for mitochondrial dysfunction and lipid accumulation. In parallel, zebrafish embryo assays provided organism-level supporting evidence. To integrate the diverse mechanistic evidence, Dempster–Shafer decision theory (DST) was applied, allowing uncertainties to be quantified and to combine different evidence collected from *in vitro* assays. Collectively, these data supported a hypothesis-driven read-across, illustrating how *in vitro* NAMs, omics profiling, *in silico* integration, and AOP-guided testing can be combined within IATA to enhance confidence in predictions for chronic toxicity and reduce reliance on animal studies (Escher et al., 2022).

However, still NAM has some of the limitations especially to predict systemic toxicity, however efforts are ongoing in different project clusters like ASPIS which is a joint collaboration of three European projects for animal-free safety assessment of chemicals. A recent survey indicated that NAMs are being used by manufacturers at the internal level, but currently they are not considered sufficiently validated to be included in the REACH regulation. Also, the time needed for acceptance of NAM is quite high with limited NAM-based test guidelines adopted per year (Bearth et al., 2025). As a general perception, NAMs are considered less safe and traditional idea about animal testing being the “gold standard” needs to be changed to improve the acceptance of NAM in the regulatory framework.

## 4 Automation of different NAMs approaches

Over past few years, machine learning (ML) and artificial intelligence (AI) has played significant role in generating high-throughput data by *in-vitro* models, toxicogenomic and *in silico* modeling. AI approaches are becoming increasingly famous for toxicity prediction as NAM for next-generation risk assessment (NGRA). Integration of AI with high-throughput data can help in identification of bioactivity pattern across thousands of chemicals supporting prioritization of compounds and grouping them for reducing the experiments. Kim et al. used ToxCast database to develop mechanism-based toxicity-prediction models. Almost 1,485 bioassay dataset was collected and used for model training with 5 ML algorithms: logistic regression, decision tree, random forest, gradient boosting tree and XGBoost providing 24,500 models for 980 assays. 311 models were selected related to endpoints like acute toxicity, carcinogenicity, endocrine disruption and developmental and reproductive toxicity providing a starting point to incorporate AI based models for NGRA using *in-vitro* data (Kim et al., 2025).

New risk assessment methods can be developed by training ML models with large biological and chemical databases which can predict molecular interaction between chemicals and how the chemical is interacting with different receptors. ML models are being developed to predict ADME properties like caco-2 cell permeability, plasma unbound fraction, clearance to later incorporate in PBK models for predicting toxicokinetic of compounds (Li Y. et al., 2024; Vallance, 2016). Different type of ML based QSAR models are being developed by researchers to prioritize compounds and reduce the experimental burden (Pore and Roy, 2024).

With increasing computational power, large language models (LLMs) with contextual learning capabilities are emerging to improve automation, and with larger datasets they can be trained and fine-tuned to reduce the experimental burden for both existing and emerging chemicals (Klambauer et al., 2023). Recent advancements demonstrate the potential of LLMs like GPT-4 in the complex process of AOP construction, showing high accuracy in identifying MIEs, KEs, and linking them to adverse outcomes, thus accelerating data identification and synthesis from vast scientific literature (Shi and Zhao, 2024). LLMs can also help bridge multidisciplinary knowledge gaps and enhance standardization in AOP development (Shi and Zhao, 2024). Similarly, the GPT-3 model has been explored for data extraction and validation in the context of NAMs and AOPs, proving effective in recognizing semantic differences and extracting specific, often challenging, parameter-related entities from full-text articles, outperforming some traditional NLP tools in these areas (Viganò et al., 2024). This capability of LLMs to rapidly learn from limited examples and generate structured output can save significant manual effort in data curation and labelling, moving towards more automated and quantitative AOP mapping (Viganò et al., 2024).

Currently, efforts are ongoing toward automation of different approached for human safety assessment. KNIME analytics platform was used to develop automated workflow by integrating PBK, dynamics model and virtual cell-based assay (VCBA) model to evaluate chemical fate, kinetics and toxicity. VCBA can be used to guide *in-vitro* experiments especially test concentrations and time point for specific endpoint which can be later combined with PBK to calculate safe exposure levels for human (Sala Benito et al., 2017). Automated ML approaches are also coming up in recent years where autoML platforms like Vertex AI, dataiku and azure were employed by the researcher to develop a model for nanotoxicity prediction. However, none of three autoML platform outperform the conventional ML models, but it is beneficial for researchers with limited knowledge on ML based predictive model development (Xiao et al., 2024). Automation is not limited to computational models, but some work is ongoing for lab automation i.e., automation for compound dilution, cell seeding, media exchange, viability assessment and so on. Lab automation was achieved for embryonic stem cells (EST) for routine applications in 96- well format, enabling evaluation of several compounds in parallel (Witt et al., 2021). Automation in cell culturing and other experimental methods can help in improving reproducibility, achieving high standardization, low material consumption and increasing the confidence by reducing manual errors. Some work is also ongoing in European project like PARC where researchers are working on automated approach combining different *in silico* tools to develop an IATA for endocrine disruption. In a recent study, effort was made to develop a pipeline combining molecular docking simulation with chatGPT to simulate pharmacokinetic prediction of Oritavancin (Fatoki et al., 2024). Overall, it helped in deciphering pharmacological profile of drug along with clinical application. Similar work can be adopted for chemical risk assessment and improved by combining lab automation with molecular docking, QSAR, PBK and pharmacodynamic (PD) which can help in enriching biological knowledge about newer and existing compounds. Combining these approaches together through LLM and making them automatized can be a step towards IATA using NAMs data, however, there remain multiple challenges, especially the quality of data and format of output parameters and completeness of training data, addressing the “black box” nature of some LLMs, managing computational costs, and overcoming token limitations for full-text processing (Shi and Zhao, 2024; Viganò et al., 2024).

## 5 Harmonization and data fairification

Harmonization and data fairification are important pillars since implementing these steps can lead to the creation of machine-readable models, improve data reporting, and hence facilitate the application of NAMs. Experimental and measurement data are necessary and essential resources for modelling, analysis, drawing conclusions, and providing recommendations or guidelines. However, it is common that the background information needed in areas such as hazard, health, environment, epidemiology or toxicogenomics, among others, is found in different sources or databases, requiring their integration, which presents great difficulties (Mattes et al., 2004; Ball et al., 2022; Rozony, 2024; Samsa et al., 2005). These databases usually present heterogeneity in terms of format differences, lacking semantic integrity of form, structure, and terminologies or definitions, and may exist in structured, semi-structured or unstructured form (Samsa et al., 2005; Sindhu and Hegde, 2017). An example of this is presented in environmental risk studies, where the influence of the short- and long-term exposure to a pollutant on human health requires the unification of data from air quality, climate, meteorology and orography databases, together with public health data reflecting the outcomes or injuries. In addition, it is worth mentioning the quality of the data, which are shown to be redundant, with noise in the measurements, lack of quantity or even missing data that it is not always clear how to fill in, leading to the use of data from other sources that are sometimes used inappropriately (Ball et al., 2022). The difficulties increase in the current generation, where data grows exponentially and dynamically, which on the one hand, makes it difficult to analyze in real time and on the other hand, complicates its storage, availability, and use due to its large size.

Given the variability of the base data sources, there is a notorious need for harmonization in order to obtain standardized and quality data (Mattes et al., 2004; Ball et al., 2022). The difficulties present in this step in terms of low data quality, lack of standardization, and heterogeneity imply the need to involve experts who, on many occasions, must possess extensive knowledge in disparate fields such as database programming and manipulation, data analysis, and model development and simulation. This is where data science takes importance, which can notoriously help to reduce the amount of data and combine information from different domains into a single, reliable base from which to perform subsequent analyses, especially when manual manipulation is becoming impossible, given the large volume of real-time data. This requires novel methods for information manipulation and cleaning, and for this purpose NAMs present many advantages in extrapolation, data analysis and decision making. However, in the field of human health assessment, among others, data generated with NAMs are not performed or reported in a standardized way. Contrary to this, animal research benefits from established guidelines that simplify these aspects, as indicated in some reviews, which is a prerequisite for the implementation of alternative methodologies for risk assessment (Schmeisser et al., 2023). The use of these guidelines in human health would facilitate the extraction and interpretation of results and conclusions on the quality and usefulness of the information, as well as replicability. In this area, harmonization is even more relevant given the possibility of obtaining databases of great value and size, with information of great interest and quality, increasing the statistical power of future analyses (Cheng et al., 2024).

In the field of data processing, NAMs have strengths and limitations depending on their specific application. In terms of heterogeneity, ontology-based frameworks and tools such as schema-matching are a good option for data transformation and integration, due to their ability to standardize and structure semantics, defining terminological relations. However, it presents difficulties in terms of scalability, due to the need for manual intervention. In addition, schema-matching is limited in its use in large unstructured databases (Rozony, 2024). In this sense, tools such as Machine Learning (ML) or Artificial Intelligence (AI) allow reducing the manual cost by their automation capacity, thus dealing with scalability, as well as increasing data quality in their task of filtering, cleaning and searching for errors, inconsistencies or missing values, and in harmonization by the identification of patterns. However, both require a large amount of real and reliable data for the initial learning (Dinov, 2016). Moreover, another drawback is that these tools compromise data security (Rozony, 2024) and therefore present a significant limitation in the health field, given the sensitivity of these data and confidentiality, marked by the regulation of the competent institutions, which also affects their storage and use (Pezoulas and Fotiadis, 2024). Big healthcare is complex due to its lack of structure and heterogeneity, requiring extensive pre-processing. In these fields, blockchain technologies are decentralized and secure, accessible in real time, and therefore of great utility in their use on sensitive data, despite the fact that scalability shows some difficulty in certain studies (Rozony, 2024).

In order to deal with all the aspects mentioned above, there are studies that propose systems based on standardized models in terms of syntax and semantics, facilitating the use by other researchers (Horsburgh et al., 2009). Others have focused on the development of technological frameworks and platforms, such as standardized ecosystems for environmental sensor data manipulation that integrate data visualization, manipulation, collection, storage and modelling, in web-friendly formats (Borges et al., 2018; Bumberger et al., 2025). Thus, it can be seen that the multiple new methodologies present advantages and limitations. A key requirement is the uniformity in syntax, vocabulary, and format to ensure consistency. Additionally, the ability to process large datasets rapidly, and with confidentiality is essential to adhere to the FAIR (Findable, Accessible, Interoperable, Reusable) principles and use standardized interfaces (Ali et al., 2023; Kush et al., 2020; Pezoulas and Fotiadis, 2024). Open data sharing through platforms such as GitHub and Open Science Framework (Dinov, 2016) enables broader access for researchers. This is especially important in those domains where up to 80% of the total time is spent on data preprocessing (Huber et al., 2021).

To make the data and model machine-readable, ontology can be used, which integrates symbolic knowledge representations with text mining techniques to enhance the extraction and categorization of information from unstructured text data. Unlike traditional text mining, which relies heavily on statistical methods, it utilizes predefined ontologies to provide a structured framework for interpreting the semantic content of text.

Currently, there are multiple life-science related ontologies that can help in providing harmonized vocabulary and construct models by embedding ontology, especially to learn features for machine learning models (Kulmanov et al., 2021). Currently, there are more than 800 ontologies on the BioPortal to describe biomedical and biological entities. For instance, while building machine learning models, ontology is playing a role in predictions like drug-target protein function, protein-protein, gene-disease association, and other biologically relevant relationships. Gene ontology is one of the biggest ontologies which includes findings from more than 180,000 papers representing almost 1,000,000 annotations, providing a comprehensive model of biological systems (Gene Ontology, 2023). Recognizing the current lack of ontologies for many NAMs like PBK modeling and AOPs, multiple partners in the European Partnership for the Assessment of Risks from Chemicals (PARC) are actively developing solutions. For instance, partners within PARC’s Work Package 7 (WP7) recently developed PBPKO (pbpko). This new ontology and supporting tools are tailored for annotating PBK models to enhance their FAIRification.

AOP ontology was initially used in 2017 by Lyle Burgoon, with the motivation of developing of artificial intelligence approach to predict the adverse outcome of chemicals based on chemical screening data from high-throughput assays. This approach utilizes computational reasoners using first order logic to infer potential adverse outcome from specific chemical. The AOP Ontology organizes AOPs as a parent class with child groupings, including Developmental Toxicology AOP, Disease AOP, Liver Toxicity AOP, and Reproductive Toxicology AOP. Each grouping contains specific AOPs, such as NeuralTubeDefect and DiabetesMellitusType2. Specific instances, like “AOP Neural Tube Defect via Hoxb1,” are developed from these AOPs.

Within Work Package 7 of the PARC initiative, partners are collaborating with international experts to further refine the AOP ontology. The goal is to create a comprehensive AOP-ontology covering the entire domain, which will significantly simplify annotation processes and improve machine-readability. Overall advancing domain ontology represents a crucial step towards data harmonisation and developing more reproducible and robust predictive models. Ultimately, this refined biological domain ontology will be instrumental in advancing NAMs that support IATA.

## 6 Conclusion

Overall, this paper discusses about the different approaches which are or can become a part of NAMs. Integration of multiple techniques to extract useful information is one of the goals of IATA and other regulatory frameworks for risk assessment. This paper illustrates the scope and challenges in the existing frameworks and the level of complexity required to develop them including the approaches to increase their regulatory acceptance. Complete transition from *in-vivo* to *in-vitro* is still not possible nor is intended, since there could be challenges in translation of different output data into meaningful human or environmental risk assessment. Instead, a multifaceted integration of *in vitro*, *in silico*, omics-based, and data-driven approaches is required, with ongoing challenges in translating diverse outputs into meaningful human and environmental risk assessment. For some frameworks to convert into NAMs, data harmonization and processing steps are required. Inclusion of ontology and training large language models can help in accelerating the process of risk assessment; however, a major bottleneck remains the quality and amount of data required to perform this task.

## Glossary

ADMET: Absorption, Distribution, Metabolism, Excretion, and Toxicity; pharmacokinetic/toxicokinetic properties of chemicals
AEP: Aggregate exposure pathway
AI: Artificial intelligence
AN: Artificial neural network
AOP: Adverse outcome pathway; sequence of linked biological events leading to an adverse health or ecotoxicological effect
BMD: Benchmark dose; dose–response modeling approach used to derive PoD
BPA: Bisphenol A; chemical known for its endocrine disrupter property
CNN: Convolutional neural networks
CNS: Central nervous system
DFT: Density functional theory; quantum chemistry method for predicting electronic structure of molecules
DILI: Drug induced liver injury
ECHA: European chemicals agency; EU regulatory authority managing chemical safety under REACH
EFSA: European food safety authority; EU regulatory agency for food and chemical safety
FEL: Free energy landscape; representation of conformational states in molecular simulations
LD50: Lethal dose 50
LLM: Large language model
ΔG: Total binding energy
GCCP: Good cell and tissue culture practices
HOMO/LUMO: Highest occupied molecular orbital/Lowest unoccupied molecular orbital; descriptors of molecular stability and reactivity
HepG2: A cell line exhibiting epithelial-like morphology that was isolated from a hepatocellular carcinoma of a 15-year-old, White, male youth with liver cancer
IATA: Integrated approaches to testing and assessment; framework combining multiple data sources including NAMs
IC50: Inhibitory concentration 50
IVIVE: *In vitro* to *in vivo* extrapolation; translation of *in vitro* test data into *in vivo* exposure estimates
KE: Key events
LOAEL: lowest-observed-adverse-effect level; metric to derive PoD
MACS/FACS: Magnetic-activated cell sorting/Fluorescence-activated cell sorting
MIE: Molecular initiating events
ML: Machine learning
MM/PBSA and MM/GBSA: Molecular mechanics methods (Poisson–Boltzmann and Generalized Born surface area) to estimate binding free energies in molecular dynamics
MOFA: Multi omics factor analysis
MPS: Microphysiological systems (e.g., organ-on-a-chip); advanced *in vitro* models mimicking organ-level functions
MSC: Mesenchymal stem cells
NAM: New approach methodologies; emerging non-animal tools and approaches for chemical risk assessment
NG-PBPK: New generation physiologically based pharmacokinetic modeling
NGRA: New generation risk assessmen
NLP: Natural language processing; AI techniques for extracting knowledge from scientific text
NMR: Nuclear magnetic resonance; technique for structural and metabolomics analysis
NOAEL: No-observed-adverse-effect level; metric to derive PoD
OECD: Organization for economic co-operation and development; develops international testing guidelines for chemical safety
OMICS: High-throughput biological data approaches such as genomics, transcriptomics, proteomics, and metabolomics
OORF: OECD Omics reporting framework; guidelines for reporting and regulatory use of omics data
PARC: Partnership for the assessment of risks from chemicals; EU-funded project supporting NAM development
PBK: Physiologically based kinetic model; similar to PBPK, often used interchangeably
PBPK: Physiologically based pharmacokinetic model; computational model simulating chemical ADME in organisms
PFAS: Per- and Polyfluoroalkyl substances; a group of persistent environmental chemicals of regulatory concern
PoD: Point of departure
POS: Part-of-speech
PPP1: Phosphoprotein phosphatases1
QMRF: QSAR modeling reporting framework
QSAR/QSPR: Quantitative structure–activity relationship/Quantitative structure property relationship; predictive models linking chemical structure to biological activity or toxicity
REACH: Registration, evaluation, authorization and restriction of chemicals
RF: Random forest
RNN: Recurrent neural network
SASA: Solvent accessible surface area; measure of protein surface exposed to solvent in molecular dynamics
t-PoD: Transcriptomic PoD
TWI: Tolerable weekly intake
USEPA: United States environmental protection agency.
